# Identification of tumor stemness and immunity related prognostic factors and sensitive drugs in head and neck squamous cell carcinoma

**DOI:** 10.1038/s41598-024-66196-6

**Published:** 2024-07-10

**Authors:** Zhihua Ye, Mintao Xiao, Yinping Zhang, Anfu Zheng, Duoli Zhang, Jie Chen, Fukuan Du, Yueshui Zhao, Xu Wu, Mingxing Li, Yu Chen, Shuai Deng, Jing Shen, Xinyi Zhang, Qinglian Wen, Junkai Zhang, Zhangang Xiao

**Affiliations:** 1 Department of Medical Oncology Center, Zhongshan People’s Hospital, Zhongshan, Guangdong China; 2 Laboratory of Molecular Pharmacology, Department of Pharmacology, School of Pharmacy, Southwest Medical University, Luzhou, Sichuan China; 3 Cell Therapy and Cell Drugs of Luzhou Key Laboratory, Luzhou, Sichuan China; 4 South Sichuan Institute of Translational Medicine, Luzhou, Sichuan China; 5 School of Data Science, The Chinese University of Hong Kong, Shenzhen, China; 6 Department of Radiation Oncology, Cancer Center, West China Hospital, Sichuan University, Chengdu, Sichuan China; 7 Department of Pharmacology, School of Pharmacy, Sichuan College of Traditional Chinese Medicine, Mianyang, 621000 Sichuan China; 8 Gulin Traditional Chinese Medicine Hospital, Luzhou, China

**Keywords:** HNSCC, Tumor stemness, Sensitive drugs, HLF, CCL11, Cancer stem cells, Head and neck cancer, Immunotherapy

## Abstract

The presence of cancer stem cells (CSCs) contributes significantly to treatment resistance in various cancers, including head and neck squamous cell carcinoma (HNSCC). Despite this, the relationship between cancer stemness and immunity remains poorly understood. In this study, we aimed to identify potential immunotherapeutic targets and sensitive drugs for CSCs in HNSCC. Using data from public databases, we analyzed expression patterns and prognostic values in HNSCC. The stemness index was calculated using the single-sample gene set enrichment analysis (ssgsea) algorithm, and weighted gene co-expression network analysis (WGCNA) was employed to screen for key stemness-related modules. Consensus clustering was then used to group samples for further analysis, and prognosis-related key genes were identified through regression analysis. Our results showed that tumor samples from HNSCC exhibited higher stemness indices compared to normal samples. WGCNA identified a module highly correlated with stemness, comprising 187 genes, which were significantly enriched in protein digestion and absorption pathways. Furthermore, we identified sensitive drugs targeting prognostic genes associated with tumor stemness. Notably, two genes, HLF and CCL11, were found to be highly associated with both stemness and immunity. In conclusion, our study identifies a stemness-related gene signature and promising drug candidates for CSCs of HNSCC. Additionally, HLF and CCL11, which are associated with both stemness and immunity, represent potential targets for immunotherapy in HNSCC.

## Introduction

Head and neck squamous cell carcinoma (HNSCC) is the 6th most common malignancy worldwide with an increasing incidence^1,2^. HNSCC develops from mucosal epithelial cells of the oral cavity, pharynx, larynx and is one of the most common malignant tumors of the head and neck^3^. Diagnosis reveals that most patients with HNSCC are advanced with no history of prior malignancy, and their mortality rate can be as high as 40–50%^4,5^. The development of HNSCC is closely associated with the complexity of the immune microenvironment, leading to a high degree of malignancy and poor prognosis. The tumor microenvironment (TME) encompasses diverse cellular components, wherein immune cells play pivotal roles in tumor progression and therapeutic response. Key immune cell populations within TME include T cells, B cells, natural killer cells, tumor-associated macrophages, dendritic cells, and myeloid suppressor cells. These immune cells interact through an intricate network of cytokines and signaling molecules that significantly impact the progression and prognosis of HNSCC^6^.

Initially, risk factors for HNSCC mainly included smoking and alcohol consumption, but in the last two decades, Human Papilloma Virus (HPV) and Epstein Barr Virus (EBV) were increasingly found to influence the development of HNSCC^7,8^. The main treatments for HNSCC are surgical resection, chemoradiation or chemoradiotherapy (CRT) and immunotherapy^9^. Surgical resection is generally used for HNSCC occurring in the oral cavity^10^. CRT is the most common treatment for HNSCC, while the combination of immunotherapy and CRT is still under clinical investigation^11^. In recent years, significant advancements have been made in the field of immunotherapy for the treatment of HNSCC. Immune checkpoint inhibitors, such as PD-1/PD-L1 inhibitors, have demonstrated favorable efficacy in a subset of HNSCC patients. Nivolumab and Pembrolizumab are currently considered as the primary drugs utilized for HNSCC immunotherapy. These medications enhance T cell-mediated cytotoxicity against tumor cells by alleviating immunosuppression^12,13^. However, not all patients exhibit a positive response to immunotherapy^14^, underscoring the critical importance of comprehending the immune microenvironment characteristics specific to HNSCC for optimizing treatment strategies.

Cancer stem cells (CSCs), also known as tumor-initiating cells, constitute a small subpopulation of malignant cells with greater capacity for self-renewal, metastatic spread, and resistance to therapy^15^. Stemness is defined as the potential for self-renewal and differentiation from native cells^16^, and the in vivo tumorigenicity test is the gold standard for defining tumor stemness^17^. In the past 10 years, research on CSCs has been a hot topic^18–20^. Researchers worldwide have extensively investigated the involvement of CSCs in various aspects of carcinogenesis, including tumor invasion, metastasis, drug resistance, and treatment relapse. However, further evidence is still required to advance targeted therapy specifically targeting CSCs^21^. CSCs have been shown to have a potential role in regulating immune characteristics^22^, while the molecular mechanism remians unclear. For instance, CSCs can impede immune cell function by secreting immunosuppressive cytokines like TGF-β and IL-10, thereby promoting immune evasion^23^. Moreover, in HNSCC cases, CSCs may exhibit elevated expression levels of immune checkpoint molecules such as PD-L1 which enable them to evade immune surveillance and hinder anti-tumor activity mediated by CD8^+^T cells^24^. These intricate mechanisms highlight the significance of CSCs as a target for HNSCC therapy despite their current lack of effectiveness.

In this study, differentially expressed genes (DEGs) were screened using the expression matrix of HNSCC in The Cancer Genome Atlas (TCGA) database. With these DEGs, the stemness index of each sample of HNSCC was calculated. Then, the association between stemness of tumor and clinical characteristics was calculated and genes related to stemness were identified. Subsequently, we grouped HNSCC samples based on stemness-related and immune-related genes. On the one hand, we screened for drugs targeting CSCs by stemness and survival-related genes. Among them, the grouped genes were validated by the International Cancer Genome Consortium (ICGC) database. On the other hand, we screened for genes that were highly associated with stemness, prognosis and immunity. Finally, correlation between the genes we screened and immune characteristics was determined. The identified genes not only hold potential in predicting patient outcomes, but also present promising targets for novel therapeutic interventions. A comprehensive understanding of the immune genes and their underlying mechanisms in HNSCC could significantly contribute to the development of more efficacious immunotherapy strategies.

## Materials and methods

### Data collection and R version

A total of 502 tumor samples and 44 normal samples for HNSCC were extracted from the TCGA database (https://portal.gdc.cancer.gov/). The 109 tumor stemness related genes used to calculate the stemness index were obtained from the study of Alex Miranda et al.^25^. The expression profiles of HNSCC used for validation were downloaded from the ICGC database (https://icgc.org/). 16 immune-related genes of HNSCC for consensus clustering were extracted from the study of Yidan Song et al.^26^. All R packages used in this study are based on version R4.2.0.

### Differential analysis

The “limma” R package was used to screen DEGs from normal samples and tumor samples of HNSCC. The screening threshold for DEGs was determined as |log2 fold change|> 2 and FDR < 0.05, and the FDR refers to the corrected *P*-value. Subsequently, based on the DEGs and the CSCs related genes, the stemness index was calculated for each sample through the ssgsea algorithm of the “GSVA” R package.

### Module identification

WGCNA was used to screen the gene sets that were closely associated with stemness in HNSCC. The “WGCNA” R package was used. WGCNA is a systems biology approach used to describe patterns of gene association between different samples and can be used to identify highly synergistic sets of genes^27^. The co-expression network was constructed based on the connectivity of genes which was calculated by the selected soft threshold. Then, based on the average connectivity of genes in the network, highly related gene sets are identified and different gene modules are classified. Finally, the Topological Overlap Matrix (TOM) was derived to detect correlations between modules and phenotypes. The protein interaction network of the genes in the module was constructed by STRING (https://string-db.org/). And the ClueGO plug-in and the MCODE plug-in of Cytoscape were used to screen the downstream signaling pathways and key sub-networks for the genes in the module.

### Consensus clustering

Consensus clustering of samples was performed using the "ConsensusClusterPlus" R package. We set the clustering algorithm as "K-mean" and the clustering distance as "euclidean", and we took the clustering results at K = 3 for the subsequent analysis. Principal component analysis (PCA) was used to verify the accuracy of the clustering results and it was performed with the "FactoMineR" R package. Then, the expression levels of genes based on the clustering results were visualized by heat map through the "pheatmap" R package. Gene Set Variation Analysis (GSVA) was carried out through the "GSVA", "limma" and "GSEABase" R packages.

### Immune score analysis

The immune scores for the HNSCC samples were calculated using the ESTIMATE algorithm. The “estimate” R packages was used to complete this process. The ESTIMATE algorithm uses the expression profile data to predict the stromal cell and immune cell scores, which in turn predict the content of these two cells; finally, it calculates the tumor purity inside each tumor sample^28^.

### Drug sensitivity analysis

Survival analysis was performed by univariate COX proportional risk regression analysis and was implemented with the "survminer" and "UpSetR" R packages. After survival analysis, we screened 6 prognostic factors from stemness-related genes. With these 6 genes, the prediction of sensitive drugs was performed using “readxl”, “impute” and “limma” R package. Of which, the data of drugs are from the Cancer Chemotherapy National Service Center (CCNSC) and the Developmental Therapeutics Program (DTP, http://dtp.nci.nih.gov/dtpstandard/smiles/index.jsp).

### Statistical analysis

Wilcoxon and Kruskal–Wallis tests were used to examine the significance of the differences in each sample group. The difference with *P* < 0.05 was considered to be statistically significant. To assess the prognostic value of the genes, survival analysis was performed using the Kaplan–Meier method, and the differences were determined through the log-rank test. The correlation analysis was carried out by the “Pearson” method, and the threshold was set to |R|> 0.3.

## Results and discussion

### Results

#### Determination of stemness index in HNSCC samples

**Figure 1:**
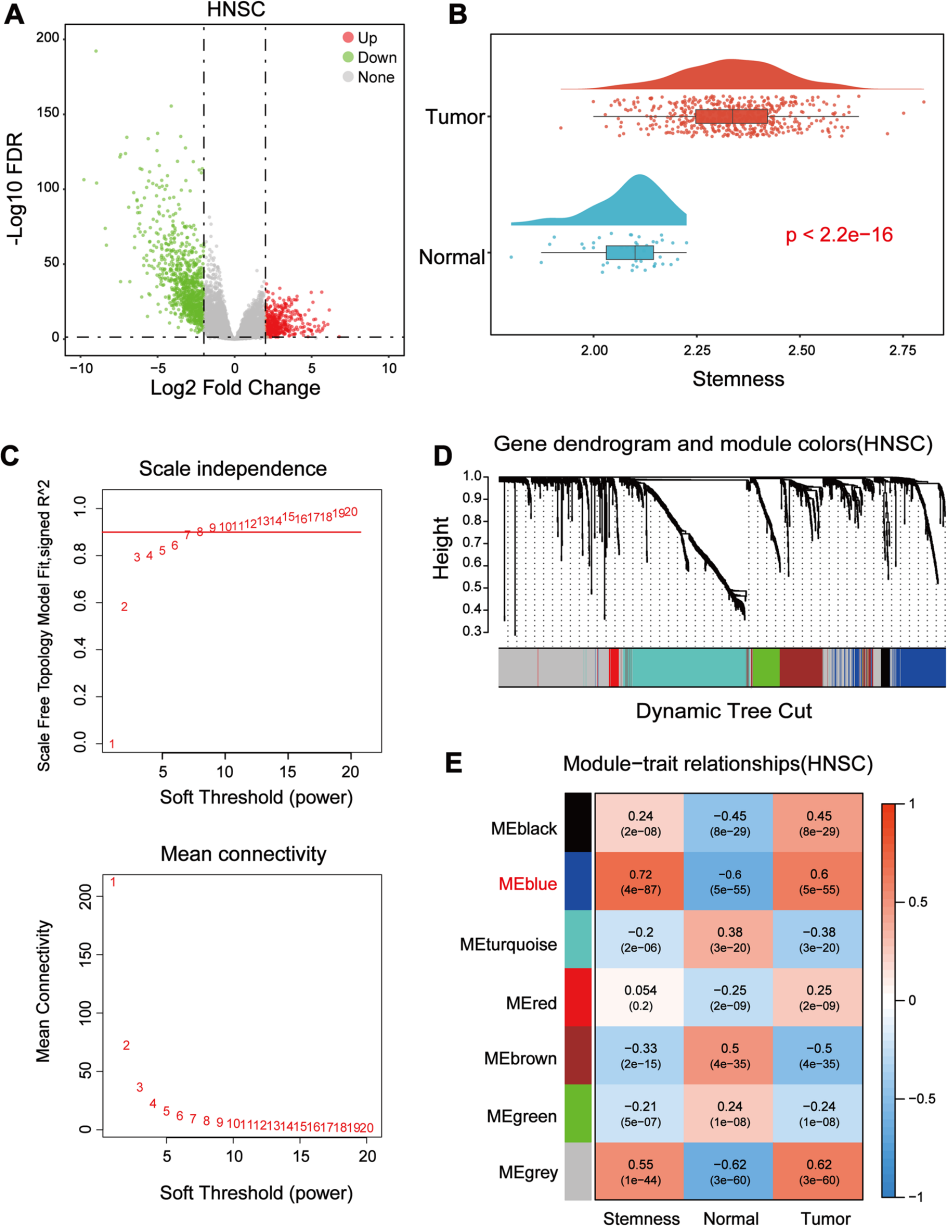
Identification of the stemness-related genes. (**A**) Volcano plot showing differentially expressed genes between the normal and tumor groups in HNSCC. FDR is the corrected P-value by Benjamini–Hochberg method. The red dots represent up-regulated genes, green dots represent down-regulated genes. Filtering condition: FDR < 0.05, |Log2 Fold Change|≥ 2. (**B**) The overall profile of stemness index in tumor/normal samples of HNSCC. (**C**) Analysis of the mean connectivity and scale independence for continuously changing soft-threshold (power). (**D**) Hierarchical clustering tree showing the 7 key modules divided based on the stemness of DEGs. (**E**) Overview of the correlation between the module and the 3 phenotypes: stemness, normal, and tumor. The color depth increases with the degree of correlation.

DEGs were obtained from differential analysis based on tumor and normal samples, as demonstrated by volcano plots (Fig. 1A). A total of 1453 differentially expressed genes were screened, including 492 up-regulated genes and 961 down-regulated genes. Using the ssgsea algorithm and 109 CSCs related genes^25^, we calculated the stemness index of each sample. The overall level of stemness index ranged from 1 to 3 and a higher level of stemness index was found in the tumor samples of HNSCC (Fig. 1B). Subsequently, association with clinical parameters revealed that both stemness index and Tumor Mutation Burden (TMB) exhibited progressive increase with tumor progression (Supplementary Fig. 1A,C). Moreover, the results of survival analysis showed that an increase in both indices was associated with a poor prognostic profile (Supplementary Fig. 1B,D).

#### Identification of key stemness related modules with for HNSCC

The 1453 DEGs and the stemness index were included in WGCNA. Firstly, the best soft threshold, also known as power, was selected by unary linear regression. The “power” is used to construct the adjacency matrix, the TOM, and to make the distribution of genes conform to the scale-free network according to the connectivity (Fig. 1C). Then, based on the calculated clustering distances, we obtained the specifics of the split modules in the network by dynamic tree cutting (Fig. 1D). Here, we get 7 modules under the filter of fixed threshold (cutheight = 0.25). Subsequently, taking stemness index and tumor/normal samples into account, we got a blue module highly associated with stemness, including 187 genes (Fig. 1E) which were referred to as stemness-related genes.

#### Identification of stemness clusters and signatures for HNSCC samples

**Figure 2:**
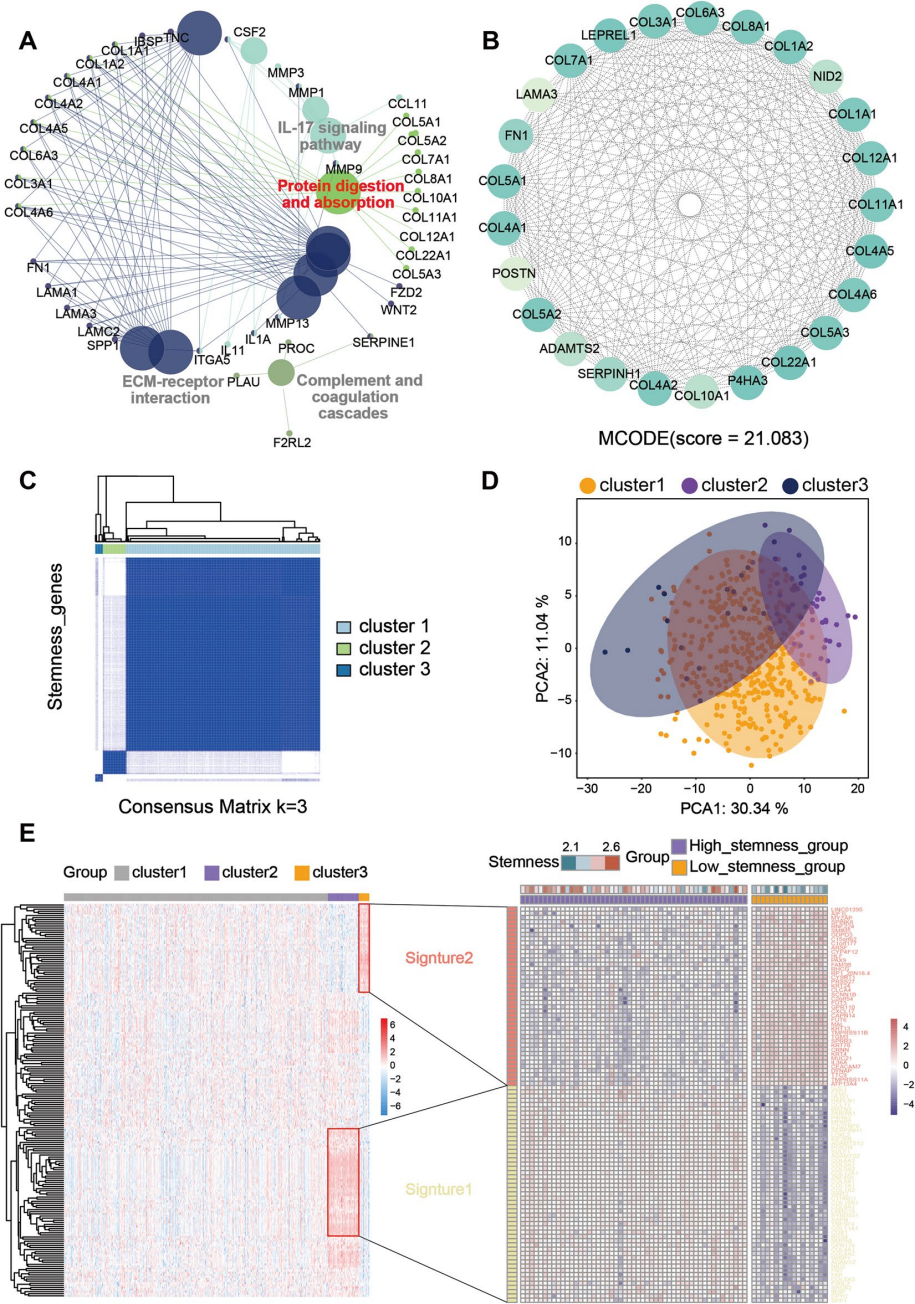
Consensus clustering of stemness-related genes for HNSCC. (**A**) Pathway enrichment of stemness-related genes. The pathways with red color indicate the most significantly enriched pathways, followed by gray color. (**B**) Key sub-networks extracted from the protein interactions network constructed from the DAVID database. The shade of green represents the level of the score, the higher the score, the darker the color. (**C**) The consensus clustering of samples according to stemness-related genes. (**D**) PCA on 3 clusters. (**E**) Expression profiles of the signature gene set corresponding to the 3 clusters. Based on the stemness index, the heat map shows that the expression of signature 2 (top) is higher in cluster 2, defined as the high stemness group (purple), while the expression of signature 1 (bottom) is higher in cluster 3, defined as the low stemness group (orange). The heat map was generated using the "pheatmap" R package.

The results of the enrichment analysis showed that the stemness-related genes were significantly enriched in the protein digestion and absorption signaling pathway (Fig. 2A). Meanwhile, we found a key sub-network by constructing a protein interaction network of stemness-related genes. The genes in the sub-network were highly similar to those enriched in the protein digestion and absorption pathway (Fig. 2B).

Based on the stemness-related genes, 3 clusters were divided by consensus clustering (Fig. 2C). Subsequently, we verified the feasibility of our grouping by the dimensionality reduction effect of PCA (Fig. 2D). Then, based on the stemness subgroups, the expression profiles of stemness-related genes were visualized by heat maps (Fig. 2E). Results demonstrated that the expression pattern of stemness-related genes in the 3 clusters were distinctly different. The expression of 2 groups of genes, signature 1 and 2, were especially high in cluster 3 and cluster 2, respectively. We compared the expression level of these genes between stenmess index high versus low groups and found that the expression of signature 1 was higher in stemnes low group while the expression of signature 2 was higher in stemness high group, suggesting that cluster 2 was relative high stemness group and cluster 3 was low stemness group.

**Figure 3:**
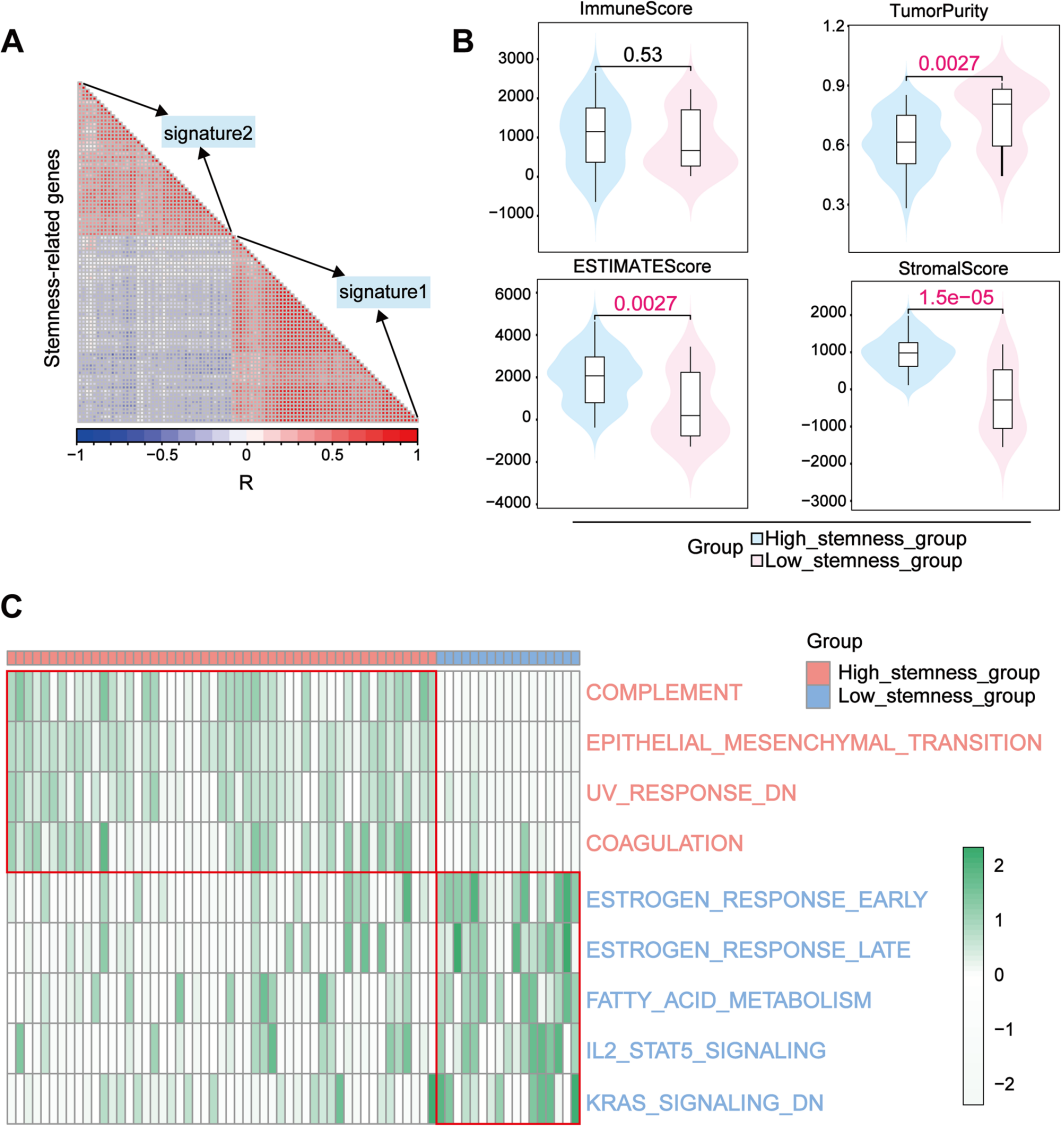
GSVA for stemness subgroups. (**A**) Correlation analysis of two signature gene sets. The red color indicates a positive correlation and the blue color indicates a negative correlation. The two gene sets are negatively correlated as shown in the plot. (**B**) The overall profile of the 4 immune scores of high and low stemness groups was calculated by the ESTIMATE algorithm. (**C**) GSVA of stemness subgroups. Red represents the high stemness group, which is significantly enriched in immune-related biological processes; blue represents the low stemness group, which is significantly enriched in biological processes related to cancer progression.

Next, we checked the correlation of genes between gene signature 1 and 2 based on the expression level of stemness related genes (Fig. 3A). The results indicated that the expression of genes in these 2 signatures were negatively correlated, which further validated our grouping results. Therefore, we included these two signatures in the subsequent analysis.

#### Relationship between immunity, biological processes and stemness

To explore the association between stemness subgroups and immunity, we tested the overall profile of 4 immune scores between stemness subgroups. Results demonstrated that the high stemness group had a higher ImmuneScore, ESTIMATEScore and StromalScore compared to the low stemness group, while the TumorPurity was lower (Fig. 3B). To find pathways related to stemness high and low subgroups, GSVA was conducted. The results showed that the high stemness group was mainly enriched in processes, including complement, epithelial-mesenchymal transition (EMT), etc. (Fig. 3C). Meanwhile, the low stemness group was mainly enriched in the biological processes related to tumor progression, including KRAS signaling, IL2-STAT5-related signaling pathways, etc. (Fig. 3C).

#### Identification of sensitive drugs targeting CSCs

**Figure 4:**
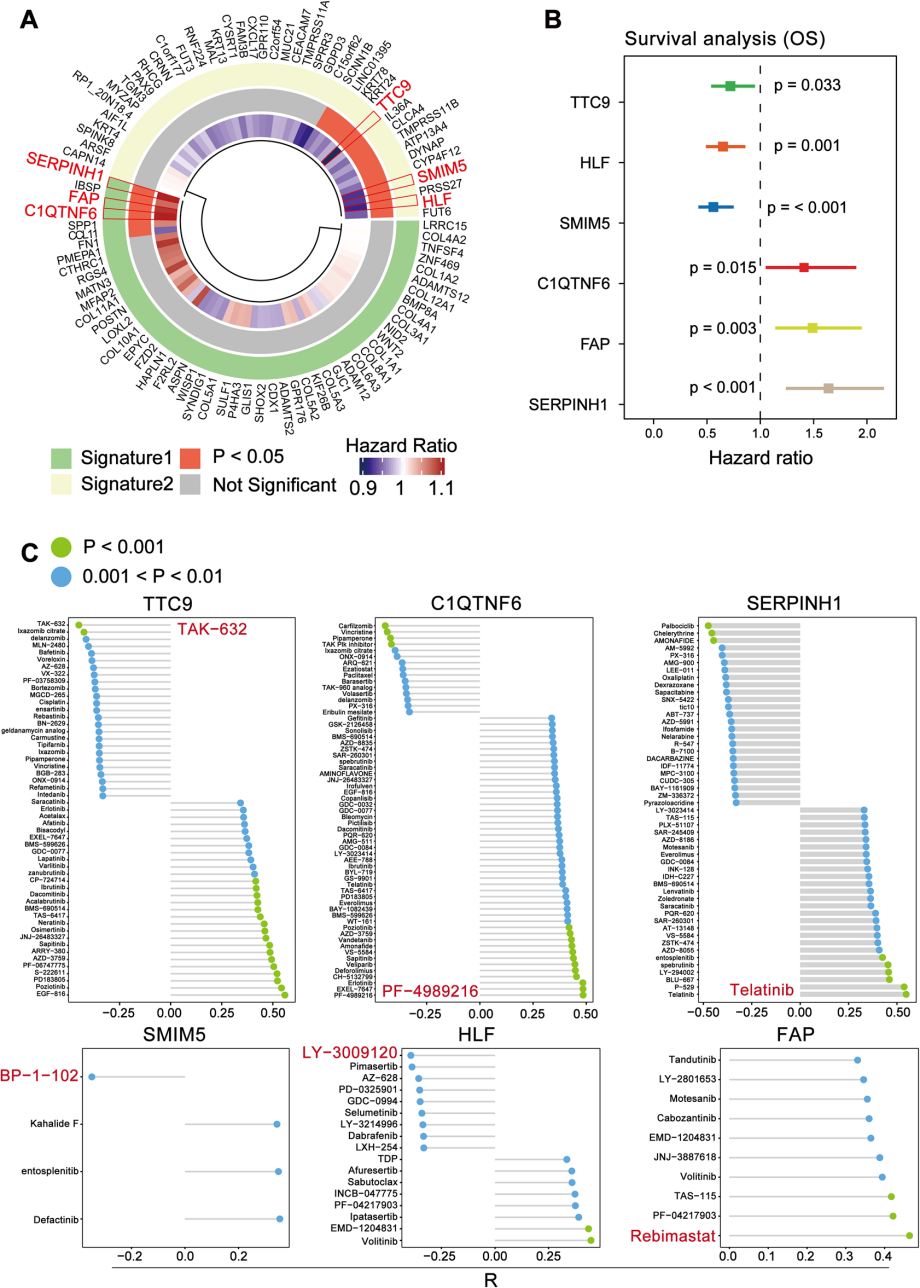
Identification of drugs targeting HNSCC CSCs. (**A**) The cyclic heat map, generated using the "ComplexHeatmap" R package, shows the results of the univariate COX regression analysis. In the outermost circles, green and yellow represent the marker genes signture1 and signture2 corresponding to the high stemness group and low stemness group. In the middle circles, red means statistically significant(*P* < 0.05), and gray means not statistically significant (*P* > 0.05). In the innermost circle, the color gradient from purple to red indicates a change from a good to a bad prognosis. The genes marked in red are the 6 genes most significantly associated with prognosis. (**B**) Survival analysis carried out by Kaplan–Meier analysis was used to verify the prognosis of the 6 genes and visualized by forest plots. Different colors represent different genes, with TTC9, HLF, and SMIM5 having a good prognosis and C1QTNF6, FAP, and SERPINH1 having a poor prognosis. (**C**) Lollipop plot visualizes drugs with sensitivity to stemness-related genes, generated using the "ggpubr" R package. Green circles represent drugs with *P* < 0.001 and blue circles represent drugs with 0.001 < *P* < 0.01. The horizontal coordinate indicates the correlation.

We selected 6 stemness-related genes associated with prognosis through univariate COX proportional risk regression analysis from the 2 gene signatures (Fig. 4A). These 6 genes included 3 genes associated with good prognosis (TTC9, HLF, SMIM5) and 3 genes with poor prognosis (C11TNF6, FAP, SERPINH1). Overall survival analysis then verified the prognostic profile of the 6 genes (Fig. 4B). Meanwhile, we verified the prognostic value and differential expression of these genes in different stemness subgroups with data from the ICGC database (Supplementary Fig. 2). In addition, C11TNF6, FAP and SERPINH1 were highly expressed in HNSCC compared to normal tissue using data from HPA database (Supplementary Fig. 3A). Next, we predicted drugs targeting the 6 genes by drug sensitivity analysis. The IC50 (the half-inhibitory concentration) of some drugs was significantly correlated with the expression levels of the genes (Fig. 4C). Drugs for which the IC_50_ was most significantly and negatively correlated with genes predicting good prognosis included TAK-632, BP-1-102 and LY-3009120. Meanwhile, for genes predicting poor prognosis, there are sensitive drugs including PF-4989216, Telatinib and Rebimastat.

#### Identification of immune subgroups for HNSCC samples

**Figure 5:**
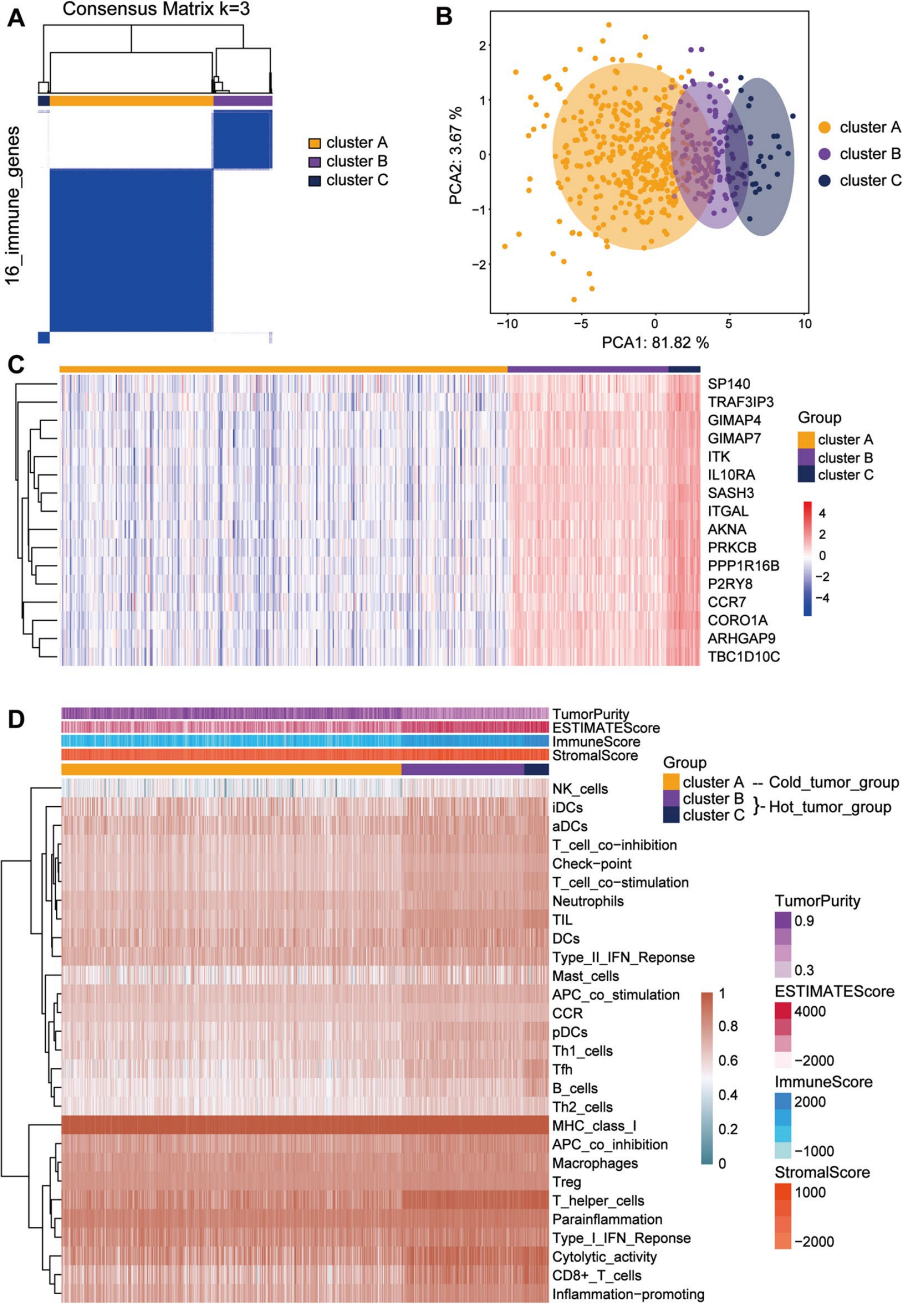
Consensus clustering of the immune-related genes for HNSCC. (**A**) Based on genes associated with immune to HNSC, 3 clusters of TCGA samples were divided by consensus clustering. (**B**) PCA of 3 immune subgroups. (**C**) The heat map, generated using the "pheatmap" R package, shows the stepwise increasing expression levels of immune-related genes in the immune subgroup. The blue gradient to red indicates a gradual increase in expression level. (**D**) The relationship between the immune subgroups and the 4 scores obtained by the ESTIMATE algorithm and the level of immune infiltration obtained by the ssgsea algorithm was visualized by heat map, also generated using the "pheatmap" R package. We can see that there is a clear demarcation between cluster A and cluster B and C. Thus, we redefined the immune subgroups. We defined cluster A as the cold tumor group and cluster B and C as the hot tumor group.

To further clarify the relationship between stemness-related genes and immunity, we identified immune subgroups through consensus clustering based on the identified immune-related genes of HNSCC (Fig. 5A)^26^. The results of PCA demonstrated unique expression pateern of immune-related genes among the 3 groups (Fig. 5B). Therefore, we examined the expression profile of 16 immune-related genes based on the immune grouping . As shown in Fig. 5C, the expression levels of the 16 genes all exhibited gradual increase from cluster A to cluster B and C. In addition, we examined the stemness index in the immune subgroups (Supplementary Fig. 3B). Meanwhile, the results of survival analysis showed that there were significant differences in overall survival among the 3 immune subgroups (Supplementary Fig. 3C). Cluster C with the lowest stemness index showed the best survival probability while cluster A with the highest stemness index had the shortest survival time. Subsequently, we calculated the degree of immune cell infiltration for each sample using the ssgsea algorithm and visualized the overall profile of immune infiltration in the 3 subgroups by heat map (Fig. 5D). The results showed that there was a gradual increase in the degree of immune cell infiltration from cluster A to cluster B and C. Thus, we defined cluster A as the cold tumor group and cluster B and cluster C as the hot tumor group. Although specific immune cell types such as Tregs and macrophages did not show significant differences among the clusters, the overall immune cell infiltration profile justifies the classification of cluster A as cold tumor and cluster B and C as hot tumor. This classification is based on the concept that cold tumors generally have low immune activity, whereas hot tumors exhibit higher immune infiltration and activity, which can lead to a more effective anti-tumor immune response despite the presence of suppressor cells. Studies have shown that hot tumors are often associated with better responses to immunotherapy ^29,30^.

#### HLF and CCL11 are highly correlated with both stemness and immunity

**Figure 6:**
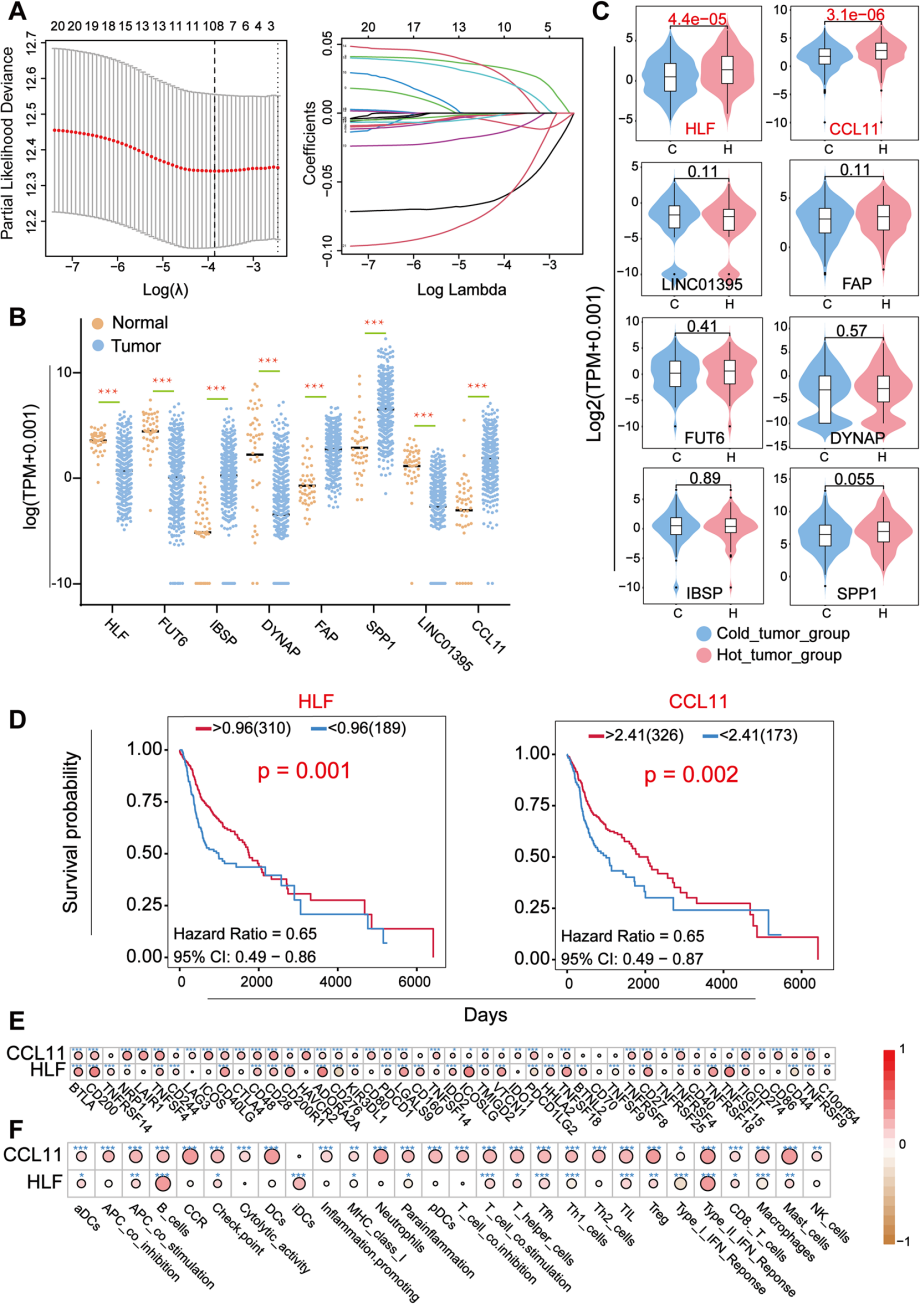
Screening of prognostic genes based on the immune subgroups. (**A**) The results of LASSO regression analysis showed that 8 genes were retained at the optimal penalty coefficient (λ). (**B**) The 8 genes were significantly differentially expressed in both tumor and normal subgroups. The brown dots represent normal samples and the blue dots represent tumor samples. (**C**) Based on the immune subgroups, we examined the overall expression of the 8 genes. However, only the expression of HLF and CCL11 was statistically significant, and both were highly expressed in the medium–high immune group. (**D**) Survival analysis on HLF and CCL11 indicated that both genes had a good prognostic profile, that is, high expression and high survival. (**E**, **F**) Dot plots visualize the correlation between HLF and CCL11 with both immune checkpoints and immune cells. Red circles indicate a significant positive correlation and brown circles indicate a significant negative correlation. *P < .05, **P < .01, ***P < .001, by spearman test.

Based on the results of the univariate COX proportional risk regression analysis, we screened 8 stemness-related genes by LASSO (Least absolute shrinkage and selection operator) regression analysis (Fig. 6A). The 8 genes are HLF, FUT6, IBSP, DYNAP, FAP, SPP1, LINC01395, CCL11. Subsequently, we examined the expression levels of the 8 genes in HNSCC (Fig. 6B). The results showed that their expression levels were significantly different in both normal and tumor samples. However, only the expression levels of HLF and CCL11 among the 8 genes were significantly different in the two immune subgroups, and both of them have higher expression levels in the hot tumor group (Fig. 6C). Therefore, we concluded that HLF and CCL11 were significantly correlated with both stemness and immunity. Survival analysis revealed that high expression of these 2 genes were associated with better survival (Fig. 6D). Moreover, correlation analyses verified that the expression of HLF and CCL11 were positively and significantly correlated with several immune checkpoints and immunological infiltration (Fig. 6E,F).

### Discussion

HNSCC is a heterogeneous group of cancers that accounts for the majority of head and neck cancers^31^. Traditional treatments including surgical resection and CRT, have led to improved survival rates. Before the advent of immunotherapy, the only new drug approved by the FDA for HNSCC was cetuximab^32^. The rise of immunotherapy has improved the survival rate of HNSCC to some extent^33^. However, the presence of CSCs is regarded as one of the main causes of treatment resistance and there is a lack of targets for CSCs in cancer^34^. One of the main manifestations of CSCs in cancer progression is the progressive loss of a differentiated phenotype and the acquisition of stem cell-like features^16^. Evidence has shown that in multiple solid tumors, the number of CSCs increases with tumor progression^35^. Due to the lack of studies on the targets of CSCs for HNSCC, here we performed a comprehensive analysis of biomarkers and sensitive drugs for CSCs of HNSCC using multiple bioinformatics analyses.

In this study, we calculated the stemness index of each sample for HNSCC by ssgsea algorithm. We found that the tumor samples in HNSCC had a higher stemness index, and there was a tendency for the stemness index to increase gradually with tumor progression (Fig. 1B, Supplementary Fig. 1). This is consistent with a previous study^36^. Meanwhile, the combination of the overall profiles of stemness index and TMB on each clinical parameter further demonstrated the important role of stemness in HNSCC (Supplementary Fig. 1). We screened a set of genes closely associated with stemness in HNSCC by weighted gene co-expression network analysis (Fig. 1C–E).

The results of the pathway enrichment analysis showed that the stemness-related genes were significantly enriched in the “protein digestion and absorption” pathway (Fig. 2A). Protein is an important component of all cells and tissues in the human body and is closely associated with many diseases. Past studies have shown that TBK1 protein and its receptor protein TBKBP1 contribute to tumorigenesis when activated by growth factors rather than by innate immune mechanisms^37^. Meanwhile, in the field of CSC research, it has been discovered that EpCAM, a transmembrane glycoprotein highly expressed in epithelial tissues, undergoes cleavage and subsequently binds to beta-catenin in cancer cells. This interaction promotes gene transcription associated with CSC properties and EMT, thereby regulating metastasis and response to cancer treatment^38^. Subsequently, by constructing protein interaction networks for stemness-related genes, we found that most of the genes in their key subnetworks belong to the COL family. Notably, most of the genes enriched in this pathway of protein digestion and uptake also belong to the COL family (Fig. 2B). Genes in the COL family are mainly used to encode various types of collagen and are mainly associated with Ehlers-Danlos syndrome^39^. Of interest, COL4A1 and COL4A2 are closely linked to tumors and can inhibit tumor growth and metastasis. Reports have indicated that COL4A1 promotes the growth and metastasis of hepatocellular carcinoma cells through activation of FAK-Src signaling^40^. Meanwhile, it has also been shown that overexpression of COL4A2 is highly associated with shorter progression-free survival in hepatocellular carcinoma patients^41^.

Next, by consensus clustering, we divided 502 samples of HNSCC patients into 3 groups (Fig. 2C). Due to the specificity of stemness-related genes expressed on cluster 2 and cluster 3 (Fig. 2D), we screened out the two groups and their corresponding genes with specific expression for further analysis. Based on the stemness index, we defined cluster 2 as the high stemness group and cluster 3 as the low stemness group (Fig. 2E). the GSVA results point out that the high stemness group is significantly enriched in immune-related biological processes, while the low stemness group is significantly enriched in biological processes related to cancer progression (Fig. 3C). In the high stemness group enrichment pathway, complement plays a crucial role in the innate immune system by participating in pathogen recognition and clearance, as well as contributing to tumor immune surveillance^42^. However, research has indicated that complement activation may facilitate cancer progression through the impact of anaphylatoxins on the TME and involvement in immune invasion pathways^43^. In HNSCC, activation of the complement system might contribute to tumor growth and metastasis. Additionally, UV response is associated with DNA damage and apoptosis^44^, potentially increasing the risk of tumorigenesis caused by UV radiation exposure^45^. In addition, the EMT is considered to be an integral part of development, wound healing, and stem cell behavior^46^. The EMT process plays a crucial role in cancer, particularly in relation to the characteristics of CSCs. Tumor cells within CSC-rich subpopulations exhibit diverse aspects of EMT program activation, and EMT is also implicated in the generation of CSCs^47,48^. In HNSCC, the EMT process can confer stem-like properties upon cancer cells, enhancing their invasive and metastatic potential as well as their resistance to treatment^49^. The low stemness group exhibited significant enrichment in estrogen response pathways, both early and late stages, as well as fatty acid metabolism and IL2, STAT5, and KRAS signaling pathways. The estrogen response pathway plays a crucial role in regulating gene expression and is involved in cell proliferation, differentiation, and metabolism. Aberrant activation of the estrogen signaling pathway has been implicated in promoting tumor growth and progression^50^. Fatty acid metabolic pathways are frequently reprogrammed in cancer cells to fulfill their energy demands for rapid growth and proliferation^51^. Alterations in fatty acid metabolism can facilitate the survival and proliferation of tumor cells by providing energy, modulating signal molecules, and influencing membrane synthesis^52,53^. Moreover, IL2, STAT5, and KRAS have been shown to play very critical roles in cancer progression^54–56^. IL2 is a crucial cytokine that regulates the immune response and can enhance the proliferation and activation of T cells and natural killer cells, thereby augmenting the anti-tumor immune response in HNSCC. High expression of IL2 has been associated with increased infiltration of immune cells and an enhanced anti-tumor immune response^57^. Furthermore, studies have demonstrated that breast CSCs exhibit heightened sensitivity to the cytotoxicity induced by IL2-activated natural killer cells. This suggests that IL2 may exert its effects by influencing CSCs within the HNSCC TME, thereby further enhancing the anti-tumor immune response. STAT5 plays a pivotal role in signaling various cytokines and growth factors, regulating cell proliferation, differentiation, and apoptosis^58^. Studies have revealed high activation levels of STAT5 in HNSCC, promoting tumor cell growth and survival. Additionally, STAT5 can modulate stem cell-related gene expression and enhance CSC characteristics, thus facilitating tumor growth, invasion, EMT, and drug resistance^59,60^. KRAS is a common oncogene harboring mutations found across multiple cancer types^61,62^. In HNSCC specifically, KRAS mutations activate downstream signaling pathways such as MAPK and PI3K/AKT pathways to promote tumor cell proliferation and survival^63,64^. Moreover, KRAS activation can also reinforce EMT features and CSC properties leading to increased invasiveness and metastatic potential in tumor cells. Through these mechanisms described above involving IL2, STAT5,and KRAS not only play critical roles in HNSCC progression but may also impact prognosis as well as therapeutic responses among patients with HNSCC by modulating CSC characteristics along with the immune response within the TME.

By univariate COX regression analysis, we screened 6 stemness-related genes, TTC9, HLF, and SMIM5 with good prognosis, and C1QTNF6, FAP, and SERPINH1 with poor prognosis (Fig. 4A). Subsequently, survival analysis verified the prognosis significance of the 6 genes (Fig. 4B). In addition, the immunohistochemical results of 3 genes with poor prognosis in the HPA database confirmed their importance in HNSCC (Supplementary Fig. 3A). Related studies reported that TTC9 is an important diagnostic and prognostic biomarker for nasopharyngeal carcinoma^65^. HLF is associated with liver fibrosis and acute lymphoblastic leukemia^66,67^. In Xiaoqi Zhang’s study, SMIM5 was shown to be closely associated with oral squamous cell carcinoma and their validation results of SMIM5 were consistent with our results^68^. CIQTNF6 belongs to the CTRP family and is also known as CTRP6. C1QTNF6 has been reported to reduce the central cardiovascular necrotic zone of transplanted hepatocellular carcinoma cells by accelerating tumor neovascularization in xenograft experiments^69^. FAP (fibroblast activation protein) has been a popular therapeutic target because its expression is implicated in many pathological processes^70^. SERPINH1 is a heat shock protein that is closely linked to collagen formation^71^.

Then we predicted the chemotherapeutic response for 6 genes, which was determined by IC_50_ provided by CCNSC (Fig. 4C). For the high stemness group, the most sensitive drugs were Rebimastat, Telatinib, and PF-4989216, while the most sensitive drugs corresponding to the low stemness group were TAK-632, BP-1-102, and LY-3009120. After literature review of the drugs, we found that they are all effective against tumor, but have not been used in HNSCC ever. Therefore, the further combination of the 6 drugs in HNSCC clinics needs to be further studied.

Next, we performed consensus clustering of TCGA samples of HNSCC accoding to the expression of the immune-related genes (Fig. 5A). Here, we defined a new grouping, cold tumor group and hot tumor group, based on the expression of immune-related genes and the degree of immune cell infiltration (Fig. 5D). Then, combining the results of LASSO regression analysis of stemness related genes with the grouping by immune-related genes, we screened out HLF and CCL11 which are related to both stemness and immunity (Fig. 6A–C). Subsequently, we validated these two genes in terms of prognosis and immune, respectively (Fig. 6D–F). Both genes had a good prognostic significance and high immune relevance. The transcription factor HLF is expressed in various cell types and has been shown to play a pivotal role in the self-renewal and differentiation of hematopoietic stem cells^72^. Furthermore, abnormal expression of HLF has been observed in malignant tumors, suggesting its potential significance in tumor progression^73,74^. In our study, we found that high expression of HLF was associated with a favorable prognosis in patients with HNSCC, highlighting its crucial involvement in regulating CSC properties and the TME. CCL11 is a member of the CC subfamily of chemokine families and is a pro-inflammatory chemokine. Its main function is to actively participate in the inflammatory response and attract immune cells to the site of inflammation. A related report indicated that CCL11 is involved in tumor immune response by regulating the recruitment of eosinophils into tumors, which further confirms its favorable prognostic profile^75^. The exact nature of the relationship between HLF and CCL11 remains incompletely elucidated; however, their distinct roles in tumor immunity imply a potential synergistic function in regulating the TME and CSC properties. HLF may indirectly modulate CCL11's activity by controlling the expression of certain immune-related genes, thereby facilitating the recruitment and activation of immune cells within the TME to impede tumor progression. Further investigations are warranted to delve into the specific mechanisms underlying the interaction between HLF and CCL11 in HNSCC, aiming to unveil their prospective synergies in tumor immunity and CSC regulation.

Although this study provides a comprehensive analysis of CSC biomarkers and sensitive drugs for HNSCC through multiple bioinformatics methods, there are some limitations. Firstly, the sample data utilized in this study were obtained from publicly available databases, which may introduce sample bias and might not fully represent all HNSCC patients. Secondly, while we have identified genes and potential drugs strongly associated with HNSCC dryness through bioinformatics analysis, these findings require further validation in laboratory and clinical settings. Additionally, although the combined impact of dry index and TMB on clinical parameters holds certain predictive value, its specific mechanism necessitates further investigation. Lastly, this study primarily focuses on CSC characteristics in HNSCC while neglecting other crucial factors that could influence tumor progression and therapeutic response such as other cell types and signaling pathways within the microenvironment.

The findings of this study offer novel insights into the investigation of HNSCC CSC biomarkers and responsive drugs, thereby contributing to the advancement of therapeutic strategies targeting HNSCC CSCs. Subsequent studies can further validate the potential efficacy of the genes and drugs identified in this study for treating HNSCC, as well as explore their applicability across diverse patient populations. Furthermore, our research revealed a significant enrichment of dry-related genes in pathways associated with protein digestion and absorption, suggesting their crucial role in regulating tumor cell metabolism. Future investigations can delve deeper into elucidating the specific mechanisms by which these genes influence tumor metabolism. Lastly, our discovery of immune-related CSC genes (such as HLF and CCL11) provides valuable leads for exploring new targets in HNSCC immunotherapy, warranting further examination on their precise roles within the tumor immune microenvironment.

## Conclusion

In conclusion, we comprehensively analyzed the key modules and signatures significantly associated with stemness in HNSCC using bioinformatics analysis. Moreover, we identified sensitive drugs targeting CSCs in HNSCC. Finally, by further combining immune, we found two genes with both a high degree of stemness association and immune infiltration, namely HLF and CCL11. The result indicates that they can be used as potential prognostic biomarkers of HNSCC. The clinical significance of these genes and drugs awaits further inevestigation which might help develop immunotherapy of HNSCC.

### Supplementary Information

Supplementary Figure 1.

Supplementary Figure 2.

Supplementary Figure 3.

Supplementary Information 1.

## Data Availability

The original contributions presented in the study are publicly available. This data can be found here: https://portal.gdc.cancer.gov/, https://dcc.icgc.org/.

## Abbreviations

CSCs: Cancer stem cells
HNSCC: Head and neck squamous cell carcinoma
TME: Tumor microenvironment
EMT: Epithelial-mesenchymal transition
ssgsea: Single-sample gene set enrichment analysis
WGCNA: Weighted gene co-expression network analysis
HPV: Human papilloma virus
EBV: Epstein barr virus
CRT: Chemoradiation or chemoradiotherapy
DEGs: Differentially expressed genes
TCGA: The cancer genome atlas
ICGC: International cance genome consortium
TOM: Topological overlap matrix
PCA: Principal component analysis
GSVA: Gene set variation analysis
CCNSC: The Cancer Chemotherapy National Service Center
DTP: The developmental therapeutics program
TMB: Tumor mutation burden
IC_50_: The half-inhibitory concentration
LASSO: Least absolute shrinkage and selection operator
FAP: Fibroblast activation protein
