# Supplementary figures and images for: Identification of tumor stemness and immunity related prognostic factors and sensitive drugs in head and neck squamous cell carcinoma
Source: Sci Rep. 2024 Jul 10;14:15962. doi: 10.1038/s41598-024-66196-6 (PMC11236973; doi:10.1038/s41598-024-66196-6)

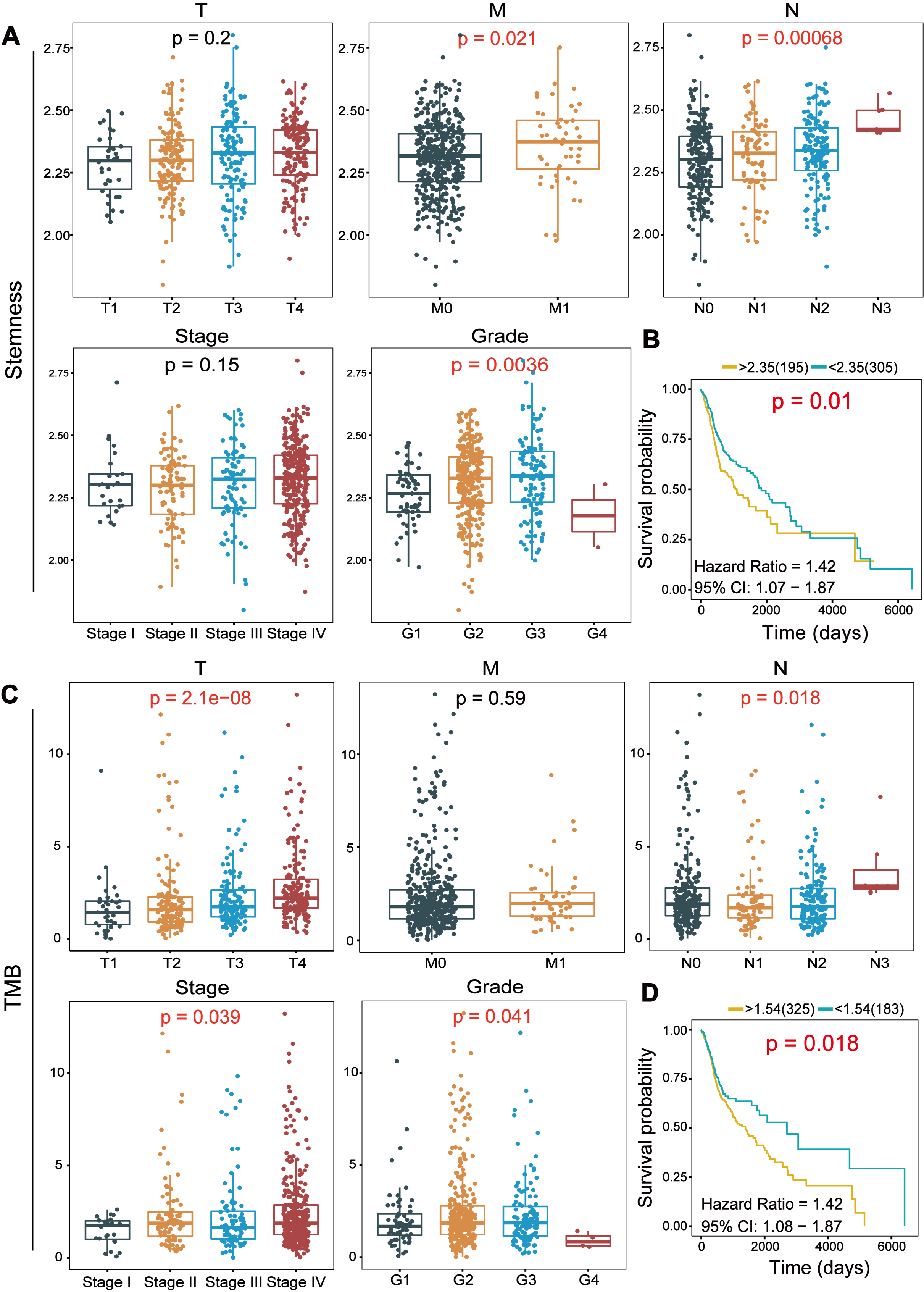

Supplement: Supplementary file 1 — Supplementary Figure 1. [file 41598_2024_66196_MOESM1_ESM.tif]

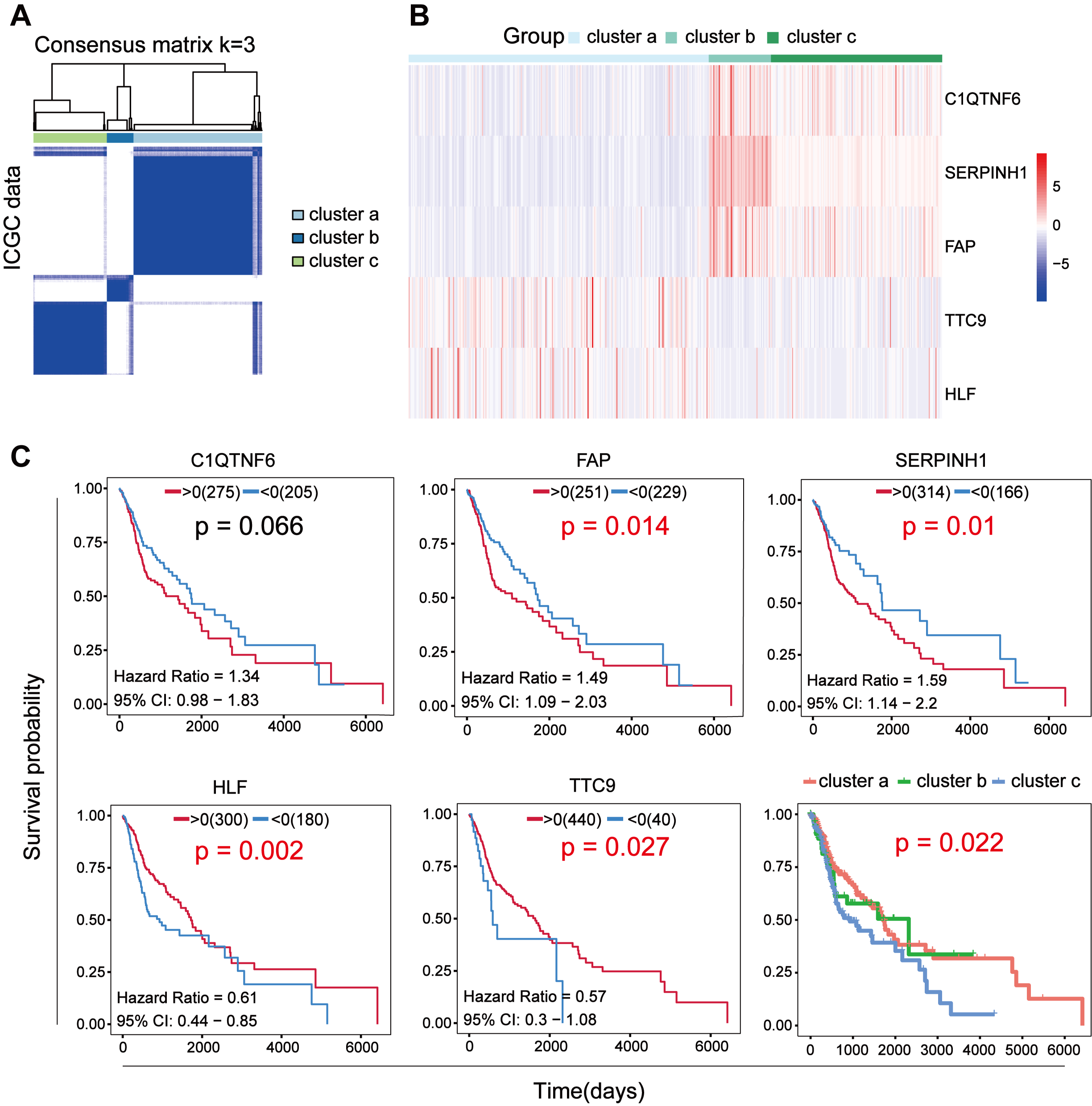

Supplement: Supplementary file 2 — Supplementary Figure 2. [file 41598_2024_66196_MOESM2_ESM.tif]

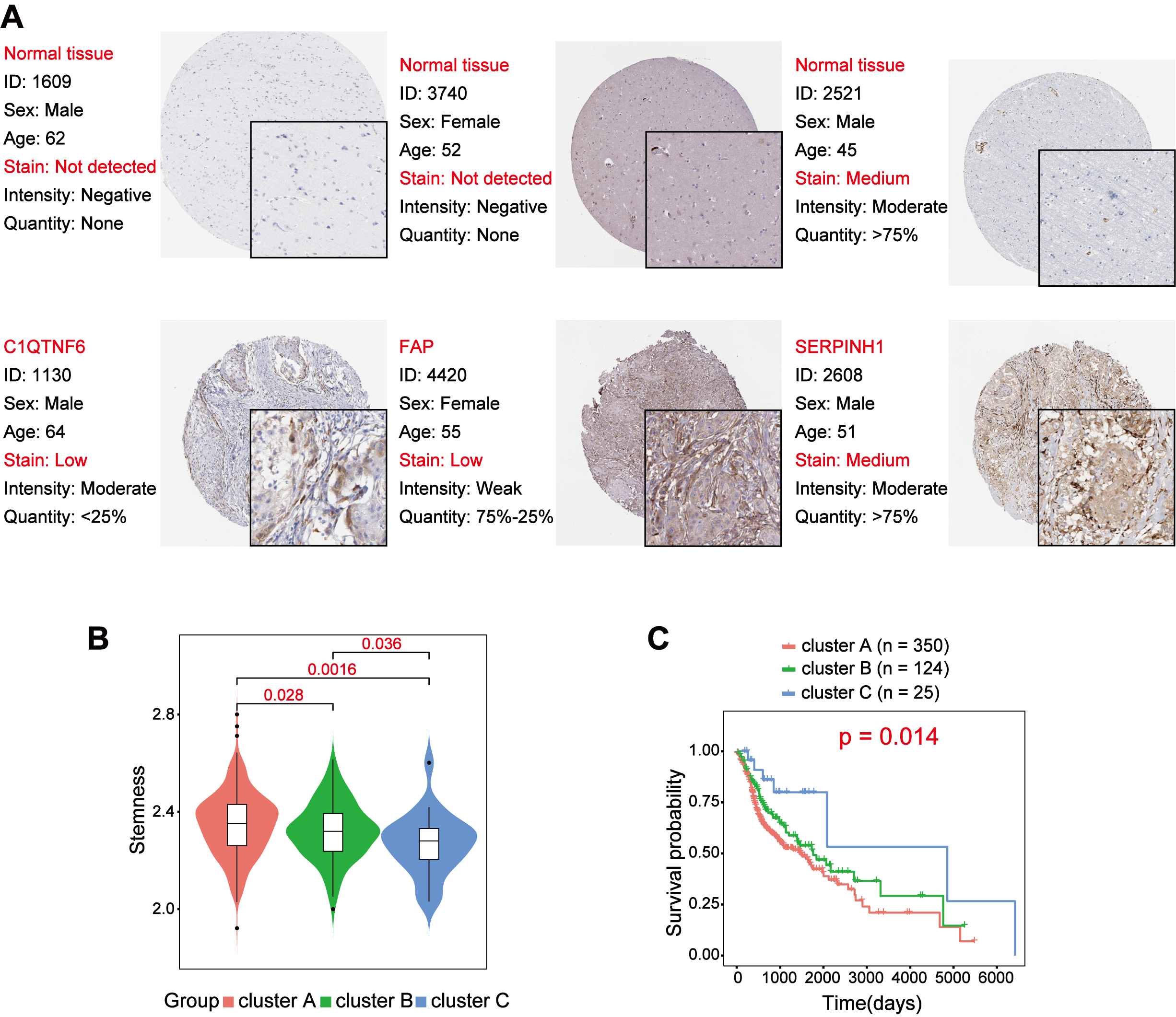

Supplement: Supplementary file 3 — Supplementary Figure 3. [file 41598_2024_66196_MOESM3_ESM.tif]
