# Supplementary material for: Identification of tumor stemness and immunity related prognostic factors and sensitive drugs in head and neck squamous cell carcinoma
Source: Sci Rep. 2024 Jul 10;14:15962. doi: 10.1038/s41598-024-66196-6 (PMC11236973; doi:10.1038/s41598-024-66196-6)
Supplement: Supplementary file 4 — Supplementary Information 1. [file 41598_2024_66196_MOESM4_ESM.docx]

# Identification of tumor stemness and immunity related prognostic factors and sensitive drugs in head and neck squamous cell carcinoma

Zhihua Ye^1†^, Mintao Xiao^4†^, Yinping Zhang^2†^, Anfu Zheng^2^, Duoli Zhang^2^, Jie Chen^2^, Fukuan Du^2,3,4^, Yueshui Zhao^2,3,4^, Xu Wu^2,3,4^, Mingxing Li^2,3,4^, Yu Chen^2,3,4^, Shuai Deng^2,3,4^, Jing Shen^2,3,4^, Xinyi Zhang^5^, Qinglian Wen^6^, Junkai Zhang^1*^, Zhangang Xiao^2,3,4,7,8*^

^1^Department of Medical Oncology Center, Zhongshan People's Hospital, zhongshan, Guangdong, China.

^2^Laboratory of Molecular Pharmacology, Department of Pharmacology, School of Pharmacy, Southwest Medical University, Luzhou, Sichuan, China.

^3^Cell Therapy & Cell Drugs of Luzhou Key Laboratory, Luzhou, Sichuan, China.

^4^South Sichuan Institute of Translational Medicine, Luzhou, Sichuan, China.

^5^School of Data Science, The Chinese University of Hong Kong, Shenzhen, China

^6^Department of Radiation Oncology, Cancer Center, West China Hospital, Sichuan University, Chengdu, Sichuan, China.

^7^Department of Pharmacology, School of Pharmacy, Sichuan College of Traditional Chinese Medicine, Sichuan Mianyang 621000, China.

^8^Gulin Traditional Chinese Medicine Hospital, Luzhou, China.

^†^ **These authors contributed equally to this work.**

*** Corresponding author:**

Zhangang Xiao, Laboratory of Molecular Pharmacology, Department of Pharmacology, School of Pharmacy, Southwest Medical University, Luzhou, 646000, Sichuan, China; E-mail: [zhangangxiao@swmu.edu.cn](mailto:zhangangxiao@swmu.edu.cn). Junkai Zhang, Department of Medical Oncology Center，Zhongshan People's Hospital, zhognshan, 528403, Guangdong, China; E-mail: [jkz1103@163.com](mailto:jkz1103@163.com).

Supplementary Material

# Supplementary Figures


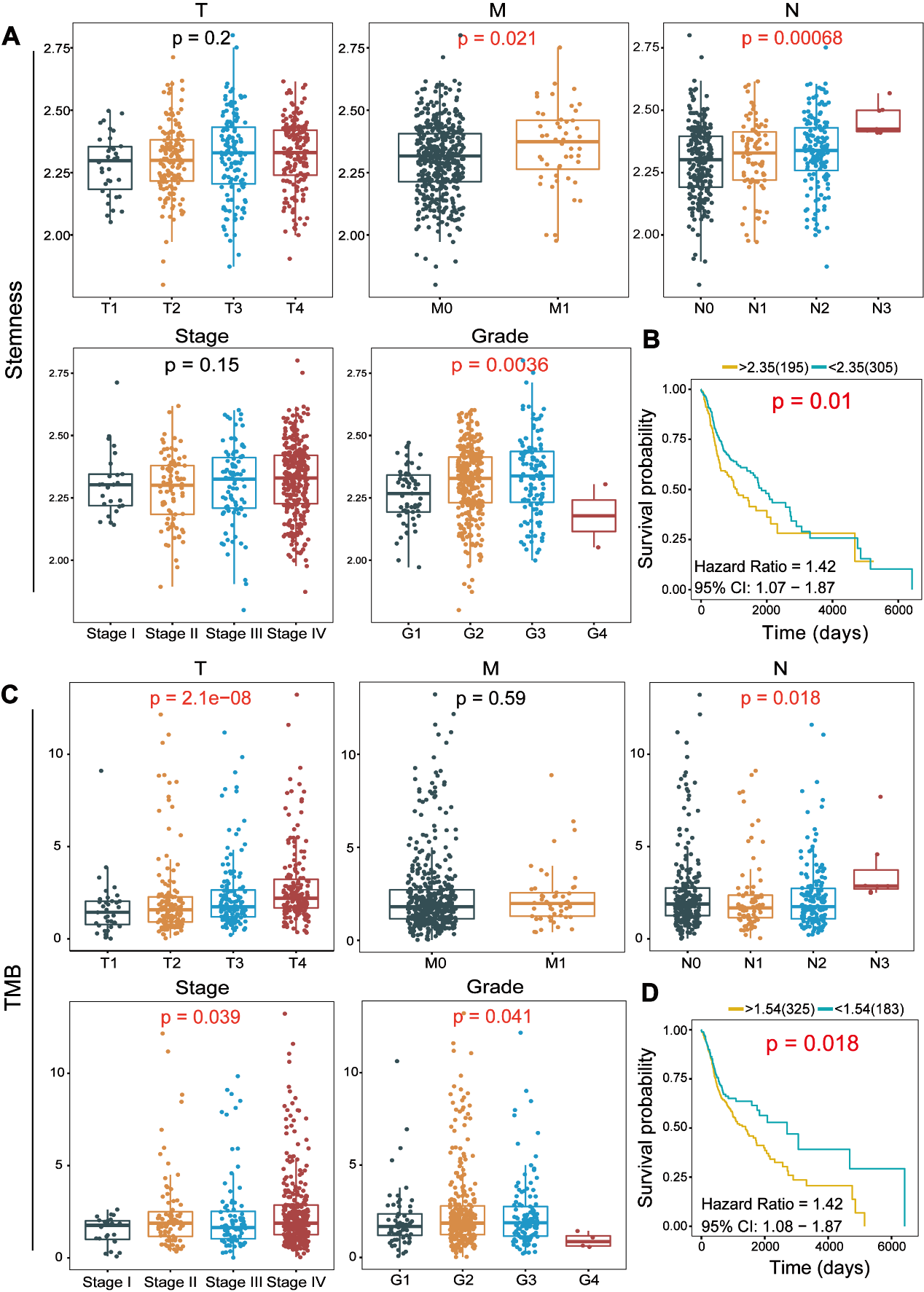


**Supplementary figure 1 Association of stemness and TMB with clinicopathological parameters.** (A, C) The overall profile of stemness index and TMB in 5 clinical parameter groupings. Both indices tend to gradually increase with tumor progression. Wilcoxon and Kruskal-Wallis tests were used to test for statistical differences. (B, D) Kaplan-Meier analysis demonstrates that higher TMB and stemness correspond to worse survival.


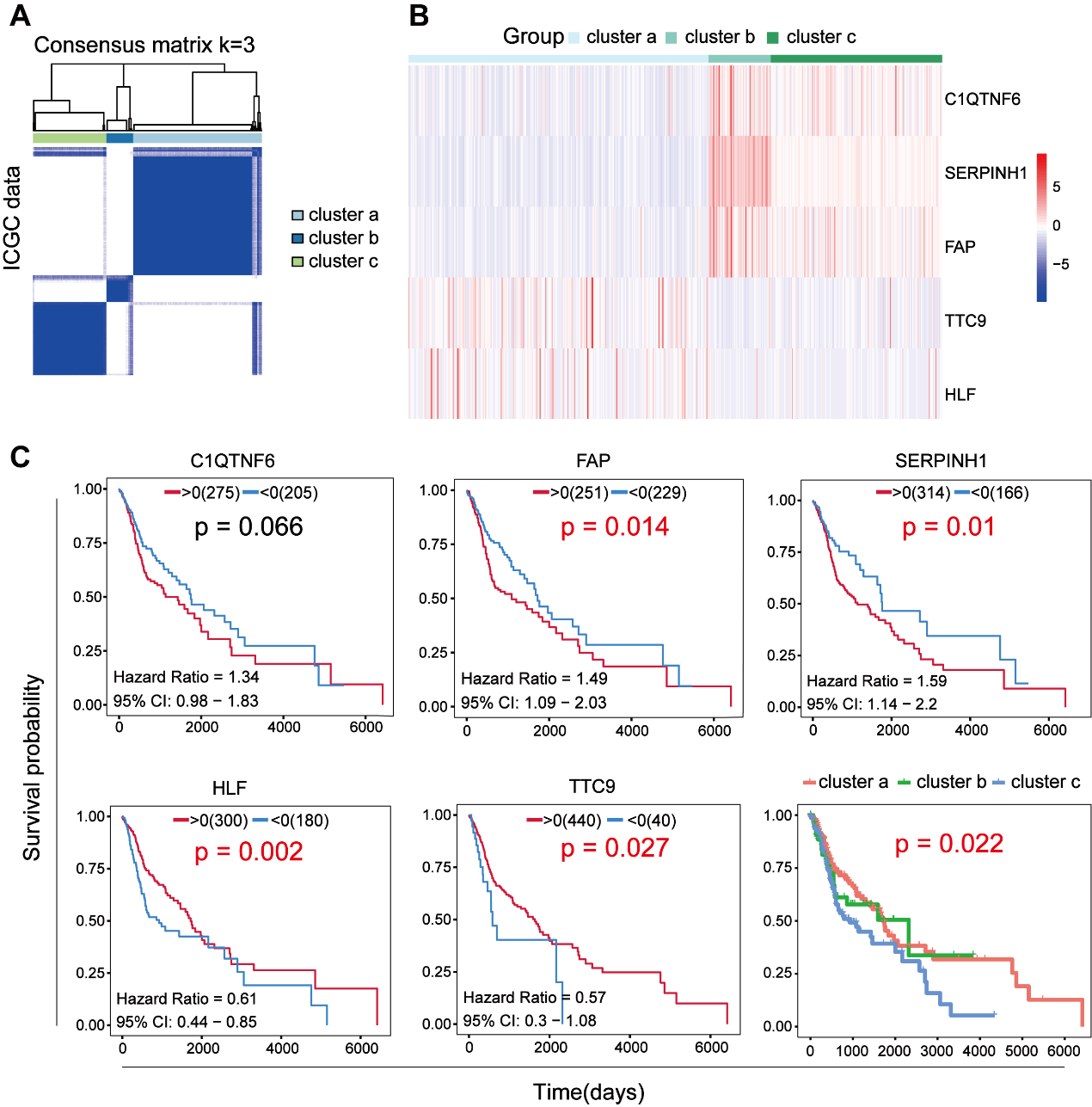


**Supplementary figure 2 Validation of the ICGC dataset.** Based on the sequencing depth of the ICGC database, we found only 5 corresponding stemness-related genes. (A) Consensus clustering of ICGC samples based on 5 stemness-related genes screened by univariate COX proportional risk regression analysis. (B) The heterogeneity of expression of the 5 genes in the grouping verified the accuracy of the grouping. The blue gradient to red indicates the incremental expression level of the gene. (C) Kaplan-Meier analysis on 5 genes exhibited the same prognostic profile as the TCGA data. Besides, the overall survival in the 3 groups was statistically significant.


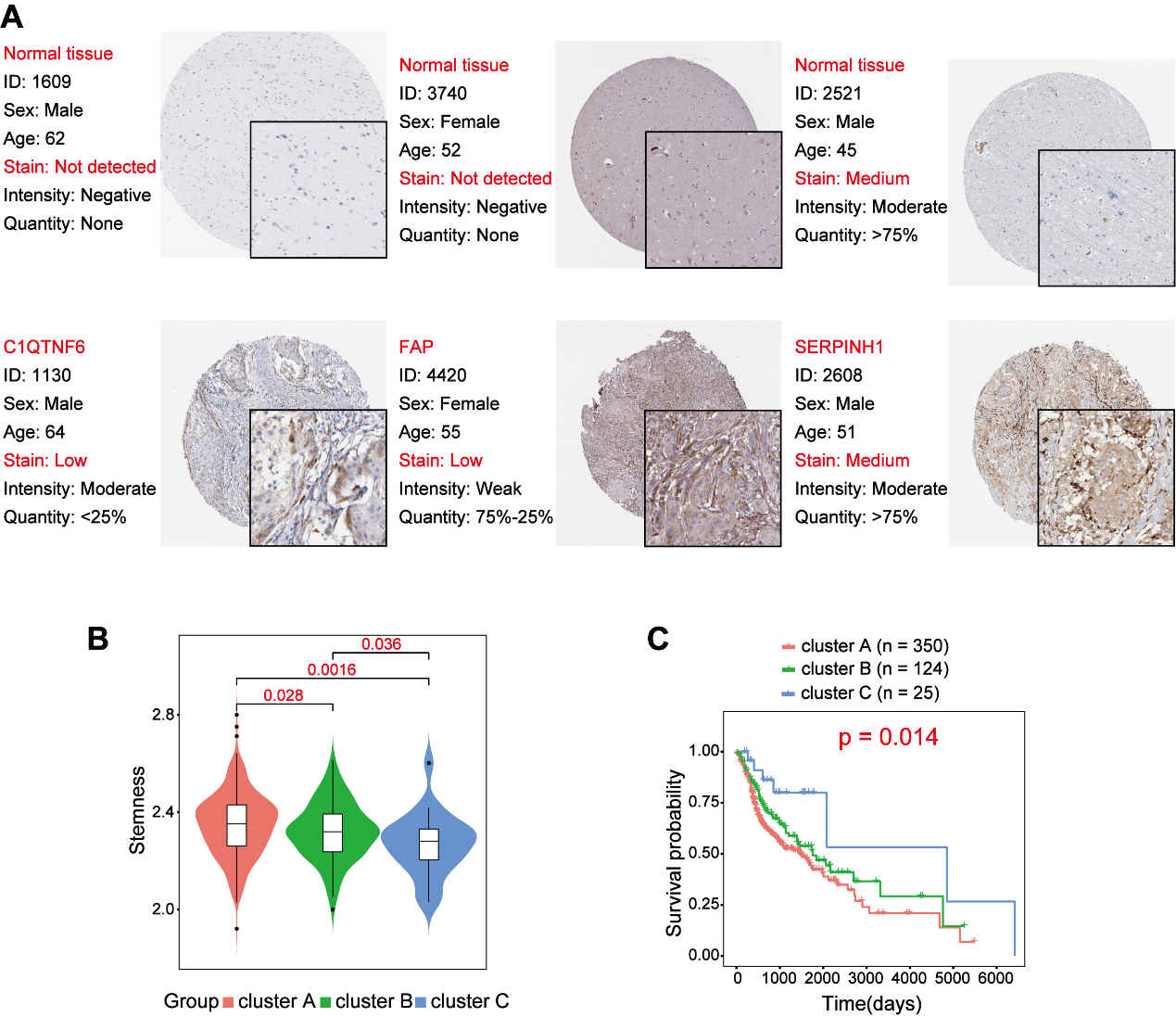


**Supplementary figure 3 Protein expression levels of C1QTNF6, FAP, SERPINH1, and general profile of immune subgroups.** (A)Tissue sections of the Human Protein Atlas were used to detect protein expression levels of C1QTNF6, FAP, SERPINH1. (B) The violin plot showed the overall profile of the stemness index in the immune subgroups. There was a stepwise decreasing trend of the stemness index in 3 subgroups. (C) The survival prognosis of 3 different clusters were statistically significantly distinctive using Kaplan-Meier analysis.
